# Pushed, Pulled, and Moored: A Systematic Review and Meta-analysis of Migration Intentions Among Medical Trainees in the Arab Region

**DOI:** 10.1007/s44197-026-00640-w

**Published:** 2026-10-02

**Authors:** Yusof Mohamed Omar, Mohamed Ashraf Elsaadany, Nour Edin Darwish, Yousuf Darwish, Lina Mohamed Omar, Mohamed Hossam Ghazy, Tasneem Deibes, Karim A. Elsodany, Jamal Ahmad

**Affiliations:** 1 Faculty of Medicine, Mansoura University, Mansoura, Egypt; 2 Faculty of Medicine, Cairo University, Cairo, Egypt; 3 College of Medicine and Health Sciences, Palestine Polytechnic University, Hebron, Palestine

**Keywords:** Physician migration, Migration intention, Medical students, Residents, Brain drain; Arab world; Health workforce, Systematic review, Meta-analysis

## Abstract

**Introduction:**

The Arab region is experiencing substantial outward migration amid persistent healthcare workforce shortages. However, reported prevalence estimates and reasons vary considerably across studies. Therefore, we aimed to estimate the pooled prevalence of migration intentions among medical trainees in the Arab region and summarize the reported drivers of migration using the Push–Pull–Mooring (PPM) framework.

**Methods:**

We searched PubMed, Scopus, Embase, and Web of Science in April 2026, supplemented by backward and forward citation searching. Studies were eligible if they reported discrete prevalence estimates of migration intentions among medical students, interns, or residents in Arab countries. The pooled prevalence was calculated using a random-effects model with a generalized linear mixed model and logit transformation. Heterogeneity was primarily assessed using Tau². Risk of bias was assessed using the Newcastle-Ottawa Scale adapted for descriptive cross-sectional studies (NOS-xs2).

**Results:**

Thirty-one studies comprising 21,614 Arab medical trainees were included. The overall pooled prevalence of migration intention was 70.7% (95% CI: 63.2%–77.2%), with substantial heterogeneity (Tau² = 0.84). Subgroup analyses were not statistically significant except for region (*p* = 0.030), with the Nile Valley reporting the highest prevalence (74.3%; k = 14) and the Arabian Peninsula reporting the lowest (41.5%; k = 3). Prevalence was numerically highest among medical students (76.0%), followed by residents (65.4%) and interns (56.7%). By intention type, general migration intent was highest (74.1%), followed by training-related (71.5%) and work-related migration (61.9%). War and insecurity (k = 10), financial insecurity (k = 9), and poor working conditions (k = 7) were the most frequently reported push factors, while higher pay (k = 11), better training opportunities (k = 10), and career growth (k = 10) were the frequently reported pull factors. Family ties (k = 6) and patriotism (k = 5) were the frequently reported mooring influences.

**Conclusions:**

More than two-thirds of medical trainees in the Arab region report intentions to migrate abroad, highlighting a major regional health workforce challenge. While intentions do not equate to actual migration behavior, the findings nevertheless support the need for health workforce policies that strengthen physician retention through investment in competitive remuneration, safe working environments, and high-quality local postgraduate training pathways while addressing broader sociopolitical drivers of migration.

**Supplementary Information:**

The online version contains supplementary material available at https://doi.org/10.1007/s44197-026-00640-w.

## Introduction

Over the past two decades, international migration of health professionals has become a defining feature of the global health workforce [1, 2]. High‑income countries’ (HICs) health systems now rely heavily on foreign‑trained physicians to fill widening service gaps, with more than half a million doctors trained abroad working in Organisation for Economic Co-operation and Development (OECD) countries by 2023—a 62% increase since 2010 [3]. This structural dependence on imported medical labour has profound implications for both the receiving and sending nations. While traditionally viewed as a brain drain/gain scenario, where the origin nation’s physician loss is the host country’s gain [4, 5], physician migration can also lead to brain waste, whereby foreign-trained physicians are unable to practice at their trained level in the destination country due to credential non-recognition, licensing barriers, and language requirements, resulting in substantial losses of time and professional potential [6]. Conversely, when regulated, this mobility can result in brain circulation, wherein origin nations benefit from the repatriation of skills upon the physician’s return, or brain exchange, where reciprocal flows between HICs produce a net-neutral or net-positive exchange of expertise [7].

The Arab world represents a particularly compelling yet understudied setting for these dynamics. Spanning 22 countries across the Middle East and North Africa (MENA), the region is internally heterogeneous. As of 2025, nine of its countries are classified by the World Bank as fragile and conflict-affected, including Iraq, Syria, Yemen, Sudan, and Libya [8, 9]; while others, such as Egypt, Jordan, Morocco, and Tunisia, face chronic fiscal constraints, political instability, and fragile health systems in absence of active conflict [10]. On the other hand, high-income Gulf Cooperation Council (GCC) states, namely Saudi Arabia, the United Arab Emirates, and Qatar, function as powerful regional magnets for medical talent [11]. This internal stratification means the Arab world simultaneously experiences brain drain from its poorer states and brain gain within its wealthier ones [11]. Beyond intra‑regional flows, the Arab region also contributes substantially to international medical graduate pools: by 2013, 14,496 physicians trained in 15 Arab countries were actively practicing in the United States, comprising 1.6% of its entire physician workforce [12]. Yet, at the same time, the WHO Eastern Mediterranean Region is projected to account for more than 20% of the estimated global shortfall of 10 million health workers by 2030, underscoring the scale of the workforce crisis facing Arab health systems [13]. Taken together, these patterns of outward migration amid deepening domestic shortages highlight the urgent need to quantify the scale of migration intent among Arab medical trainees and systematically evaluate the systemic forces driving this impending workforce attrition.

Empirical data on migration intentions among medical trainees in the region underscore the magnitude of this phenomenon. For example, approximately 89.4% of Egyptian medical undergraduates [14], 85% of Jordanian medical students [15], and 95.5% of Lebanese medical students [16] express intentions to emigrate for postgraduate training abroad. Although these figures are based on self‑reported intentions rather than observed migration, large‑scale comparative work by Tjaden et al. has shown that country‑level emigration intentions closely track actual migration flows, especially from lower-middle-income countries (LMICs) and low-income countries (LICs) to HICs [17]. In regions such as the Arab world, where comprehensive data on physician emigration are sparse or fragmented, intention surveys therefore provide a valuable proxy for underlying migration dynamics [18]. To further understand the drivers of migration intentions, researchers often adopt the Push–Pull–Mooring (PPM) framework as a lens for organizing the various economic, political, and sociocultural forces shaping migration decisions [19].

The PPM framework conceptualizes migration intentions as the net result of origin “push” forces, destination “pull” forces, and “mooring” factors that either hinder or enable movement [19]. In Arab LICs and LMICs, key push factors include chronically low remuneration, limited or unpredictable postgraduate training opportunities, and heavy workloads in under‑resourced facilities, all of which are repeatedly cited by Egyptian, Jordanian, and Lebanese trainees as primary reasons for wanting to leave [11, 14, 15, 20]. Pull factors, by contrast, centre on the prospect of substantially higher incomes, access to structured residency and fellowship programmes, better research and teaching environments, and greater personal and professional security in GCC states and Western destinations [14, 15, 21]. Mooring factors, on the other hand, operate in both directions; strong family obligations, cultural and linguistic attachment, and a sense of professional duty to serve one’s community can discourage emigration [14, 20, 22], while language proficiency [16], diaspora networks [15], and pathway‑specific schemes (e.g. scholarships or targeted recruitment programmes) [23] can lower the barriers to moving abroad and make migration more likely.

However, despite the proliferation of cross‑sectional data investigating migration intent within specific Arab nations, the current literature remains localized and lacks a regional quantitative synthesis. While existing literature has explored the movement of physicians and medical students globally, there is a lack of research specifically targeting the Arab medical trainee cohort or organizing their migration drivers through the PPM framework. These trainees constitute a unique demographic, as their aspirations to emigrate are often established prior to independent practice, heavily influenced by their academic environment and local postgraduate prospects, thus making this a critical junction for retention intervention. Against this background, and because aggregate migration intentions correlate strongly with actual subsequent migration flows [17], the primary aim of this systematic review and meta-analysis is to quantitatively synthesize the prevalence of migration intentions among medical trainees (students, interns, and residents) across the Arab region, thereby quantifying the scale of the potential physician migration crisis in the region. Secondarily, we aim to organize their reported drivers of migration utilizing the PPM theoretical framework to help inform targeted retention and policy interventions.

## Methods

We followed the recommendations of the Preferred Reporting Items for Systematic reviews and Meta-Analyses (PRISMA) guidelines while reporting this manuscript [24]. This study was prospectively registered in PROSPERO on 17 April 2026 (CRD420261372438; https://www.crd.york.ac.uk/PROSPERO/view/CRD420261372438).

### Outcome Definition

As Tjaden et al. argue [17], migration intention is an elusive term without a single clear definition. It has been described as an individual’s intention, desire, consideration, inclination, or plan to migrate or leave their home country, regardless of subsequent action [25]. In this light, and due to expected heterogeneity in outcome definitions across studies, “migration intention” in this review was defined as any self-reported desire, plan, or preference among Arab medical trainees to leave their Arab home country. Because this overarching construct encompasses distinct operational pathways, such as pursuing postgraduate training abroad, relocating for employment, or general emigration, we pre-specified a construct-stratified subgroup analysis to evaluate each dimension independently.

### Literature Search Strategy

We searched PubMed, Scopus, Embase, and Web of Science on the 18th of April 2026. The search strategy combined Medical Subject Headings (MeSH) and free-text terms covering medical trainees (e.g., medical students, interns, residents), migration intention-related outcomes (e.g., migration, emigration, brain drain, career intentions), and all Arab countries. The search syntax was adapted for each database, and the full strategy is provided in Table S1 (Additional file 1). In addition, we conducted backward citation tracking by screening the reference lists of the included studies, followed by forward citation tracking by screening the studies that cited the included articles.

### Eligibility Criteria

We included all studies that quantitatively assessed the intention to migrate among medical trainees (medical students, medical interns, and resident physicians) studying or training in any Arab country. Studies were eligible if they utilized any broad definition of migration intention, provided they reported these intentions as discrete categorical data (i.e., frequencies and percentages) to allow for the pooling of prevalence estimates. No restrictions were placed on publication language or status; preprints were eligible.

We excluded studies on practicing physicians, as well as those evaluating non-medical healthcare populations (e.g., nursing, dental, or pharmacy students). Studies on Arab medical trainees who had already emigrated and were currently training abroad were also excluded, as these represented secondary mobility rather than initial emigration intent. Studies measuring migration intention using Likert-scale or other continuous measures, rather than discrete categorical outcomes, were also excluded. Finally, qualitative studies, reviews, and editorials were excluded.

### Selection Process

After manual removal of duplicates, the titles and abstracts of all retrieved papers from database searching were screened using Rayyan software [26]. Next, the full texts of eligible studies were reviewed to determine their eligibility for inclusion in the review. The total deduplicated records were divided into two subsets, with each subset screened independently in duplicate by a dedicated pair of reviewers, ensuring that every record was independently evaluated by two reviewers. Disagreements within pairs were resolved by consensus, and persistent uncertainties were adjudicated by a fifth reviewer. Full-text articles were similarly assessed in independent duplicate. Records identified through backward and forward citation tracking followed the identical dual-independent screening and consensus protocol.

### Data Extraction and Synthesis

Four authors independently extracted data using a standardized Google Sheets form, while an independent fifth author reviewed the dataset to ensure accuracy. Extracted data included study design characteristics (author, year, sampling technique), population details (cohort type, sample size), and the reported prevalence of migration intentions. Additionally, following the same extraction method, migration drivers were mapped to the PPM framework according to how they were operationalized and framed in the original manuscripts, including the wording of questionnaire items and/or authors’ descriptions of the measured constructs, rather than through reinterpretation by the review team.

To identify potentially overlapping study populations, publications from the same countries, institutions, or study periods were compared based on participant characteristics, sample size, recruitment setting, study period, and methodological characteristics. Publications with evidence of duplicate datasets were excluded, with three publications excluded for this reason.

To address methodological heterogeneity in how migration intention was measured across the included studies, the extracted outcomes were first pooled to evaluate the overall primary outcome. Subsequently, the data were grouped into three predefined and mutually exclusive categories for subgroup analysis: (1) training-related migration (defined as the intent to travel abroad specifically for postgraduate residency, fellowship, or subspecialty training), (2) work-related migration (defined as the intent to relocate specifically for employment or to pursue a career), and (3) general migration intention (defined as broad, undifferentiated, or composite queries where specific training or work motives could not be isolated).

Furthermore, each study was mapped to the World Bank income classification [27] and Fragile and Conflict-Affected Situations (FCAS) status [8] of its host country for the corresponding year of data collection. For the single multi-country study, its overall aggregate sample was included as a single study-level estimate in the primary meta-analysis. For stratified subgroup analyses (by geographic region, country income level, and fragility status), the data were extracted at the per-country level and mapped to their respective strata without double-counting.

### Risk of Bias

Two authors independently assessed the risk of bias of the included studies. All studies were evaluated using the recently adapted simplified version of the Newcastle-Ottawa Scale (NOS-xs2) adapted for descriptive cross-sectional (prevalence) studies [28]. The NOS-xs2 evaluates studies across two primary domains: study sample selection and assessment of outcome(s). Discrepancies between reviewers were resolved through consensus or consultation with a third author.

### Statistical Analysis

All statistical analyses were performed using R [29] with the meta [26] and metafor [30] packages. The primary prevalence meta-analysis was fitted using the metaprop() function from the meta package.

The overall pooled prevalence of migration intentions was calculated using a random-effects model, employing a generalized linear mixed model (GLMM) with a logit transformation for pooling. Confidence intervals for the summary estimates were calculated using a t-distribution with k-1 degrees of freedom, an approach analogous to the Knapp-Hartung adjustment for GLMMs. Additionally, 95% prediction intervals were calculated to estimate the distribution of true prevalence rates across different settings, incorporating the estimated between-study variance (Tau²) and the standard error of the pooled estimate.

Heterogeneity among the included studies was primarily evaluated and quantified using the absolute between-study variance (Tau²), which was estimated using the maximum-likelihood estimator. Because I² estimates are artificially inflated in prevalence meta-analyses with large sample sizes (high precision), we primarily relied on Tau² to quantify heterogeneity [31].

Subgroup analyses were conducted based on seven pre-specified categorical variables registered in our PROSPERO protocol: training level, gender, geographical region, country income level, fragility status, type of migration intention, and sampling technique. For these analyses, a common Tau² was assumed across subgroup levels, which is the recommended approach to prevent unreliable between-study variance estimation in strata with modest study counts. Differences between subgroups were considered statistically significant at *p* < 0.05.

A leave-one-out sensitivity analysis was performed to evaluate the robustness of the overall pooled estimate and to identify whether any single study disproportionately influenced the summary prevalence or the overall degree of heterogeneity.

## Results

### Search Results

Our initial database search yielded 917 records, of which 377 were removed as duplicates. The remaining 540 records underwent title and abstract screening, resulting in the exclusion of 499 records. We assessed the full texts of the remaining 41 articles for eligibility, identifying 20 studies that met all inclusion criteria by reporting the discrete prevalence of migration intentions among Arab medical trainees.

**Fig. 1:**
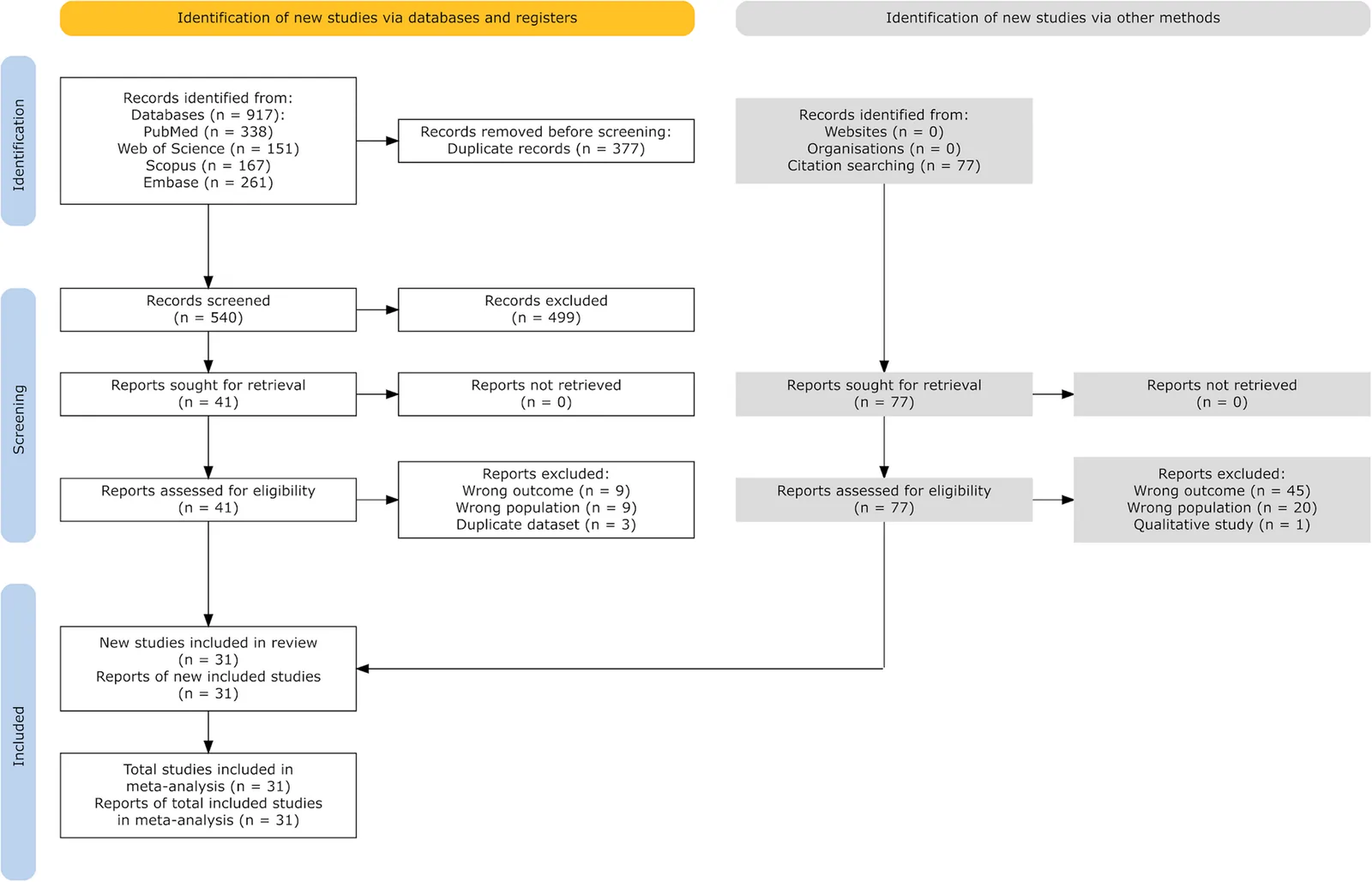
PRISMA 2020 flow diagram of study identification and selection

Subsequent forward citation searching of these articles identified 27 potentially relevant titles, of which 10 met the inclusion criteria. Finally, backward citation searching of the reference lists of these 30 studies yielded 50 additional relevant citations, one of which met the inclusion criteria. Ultimately, a total of 31 studies were included in this systematic review. Figure 1 presents the PRISMA flow diagram detailing the comprehensive study identification and selection process.

### Study Characteristics

The characteristics of the included studies are presented in Table 1. A total of 31 studies, comprising 21,614 Arab medical trainees, met the criteria for inclusion in the systematic review (Table 1).

**Table 1:** Summary of studies included in the systematic review and meta-analysis

Study ID (Author, Year)	Country (World Bank Class, FCAS)	Sampling Technique	Population	Participants’ Characteristics^1^	Migration Intention Definition	Migration Intention prevalence (%)
Abd El-Ghaffar & Abd El Salam, 2026 [32]	Egypt (LM, No)	Random	Medical students and interns	413, 54.5% female, 21.3 (2.4) mean age	Preferred future work location	51.6
Aboudeif et al., 2024 [33]	Egypt (LM, No)	Convenience	Medical students	1652, 54.1% female, 21.4 (2.2) mean age	Plan on pursuing their career abroad	52.2
Abukmail & Albarqouni, 2021 [34]	Palestine (LM, Yes)	Convenience	Medical students, interns, and residents	116, 35% female, NR mean age	Travel for specialty or subspecialty training	90.5
Ahmed et al., 2025 [35]	Sudan (L, Yes)	Convenience	Medical students	642, 61.8% female, 22.2 (2.7) mean age	Plans regarding postgraduate training	70.4
Akl et al., 2008 [16]	Lebanon (UM, No)	NR	Medical students	425, 40.9% female, 23.8 (1.4) mean age	Plan to train abroad	95.5
Al Hadhrami et al., 2024 [36]	Oman (H, No)	NR	Medical students, interns, and residents	247, 70% female, NR mean age	Preference for pursuing residency abroad	30.4
Al-Beitawi et al., 2021 [37]	Jordan (UM, No)	Convenience	Medical students	253, 56.1% female, 23.5 (0.6) mean age	Preferred country for postgraduate training	74.3
Al-Samarrai and Jadoo, 2018 [22]	Iraq (UM, Yes)	Census	Medical students	183, 59% female, NR mean age	Plan to emigrate either alone or with family	72.7
Alfadul et al., 2025 [38]	Sudan (L, Yes)	Convenience	Medical students	612, 64.4% female, NR mean age	Consider leaving country after graduation	87.1
Alfadul et al., 2026 [39]	Sudan (L, Yes)	Convenience	Medical students	4185, 67% female, 21 (NR) mean age	Consider leaving the country after graduation	87.6
Alfakhry et al., 2025 [40]	Syria (L, Yes)	Convenience	Residents	1490, 50.7% female, NR mean age	Plan to immigrate to another country	62.3
Alfiqi et al., 2026 [41]	Egypt (LM, No)	Convenience	Medical students and interns	1134, 56.7% female, NR mean age	Considering leaving country to work abroad	68.7
Alsuhaibani et al., 2018 [42]	Saudi Arabia (H, No)	NR	Medical students	150, 32.7% female, 23.3 (1.2) mean age	Intention to study abroad for specialty	64.0
Ayman et al., 2025 [43]	Egypt (LM, No)	NR	Medical students	178, 44% female, 20.94 (1.79) mean age	Wish to migrate	84.3
Barnett-Vanes et al., 2016 [44]	Iraq (UM, Yes)	Convenience	Medical students	197, 60.3% female, NR mean age	Intention to leave country after graduating	55.3
Boughzala et al., 2019 [45]	Tunisia (LM, No)	Census	Residents	68, 72% female, NR mean age	Intention to continue a specialty or work abroad	50.0
Darwish et al., 2026 [46]	Algeria, Mauritania, Syria, Egypt, Saudi Arabia (NA, NA)	Convenience	Medical students	1727, 56.5% female, 21.8 (2.0) mean age	Plan to practice or study medicine abroad	40.6
Deeb et al., 2026 [47]	Palestine (LM, Yes)	Convenience	Medical students	371, 55.5% female, NR mean age	Intention to pursue residency training abroad	59.3
Fouad et al., 2015 [48]	Egypt (LM, No)	Convenience	Medical students	940, 52.9% female, 21.2 (1.6) mean age	Intention to train abroad following graduation	80.0
Gado et al., 2025 [49]	Egypt (LM, No)	Convenience	Interns	1540, 52.3% female, NR mean age	Planned for future training outside the country	45.3
Gismalla et al., 2017 [50]	Sudan (LM, Yes)	Census	Medical students	197, 60.4% female, NR mean age	Preferred site or location of future specialty	53.3
Haouari et al., 2024 [51]	Tunisia (LM, No)	Convenience	Residents	50, 72% female, 27.7 (NR) mean age	Intention to emigrate	68.0
Kabbash et al., 2021 [14]	Egypt (LM, No)	Convenience	Medical students and residents	885, NR female, NR mean age	Intention to emigrate	89.4
Lafta et al., 2018 [52]	Iraq (UM, Yes)	NR	Medical students	418, 59.1% female, 21.9 (1.3) mean age	Plans for further training after graduation	56.9
Mohamed et al., 2015 [53]	Sudan (LM, Yes)	Random	Medical students	384, 80.2% female, NR mean age	Intention to migrate after graduation	84.6
Omar et al., 2023 [15]	Jordan (LM, No)	Census	Medical students	1006, 55.7% female, 20.8 (1.8) mean age	Intentions to leave country permanently	30.1
Sawaf et al., 2018 [54]	Syria (LM, Yes)	Convenience	Medical students	450, NR female, 23 mean age	Interested in specializing abroad	78.0
Schumann et al.,2025 [55]	Egypt (LM, No)	Convenience	Medical students	650, 49.7% female, NR mean age	Considered leaving the country and working abroad	90.8
Soqia et al., 2024 [56]	Syria (L, Yes)	Random	Residents	360, 38.4% female, NR mean age	Work location after specialization	44.7
Suboh et al., 2025 [57]	Palestine (LM, Yes)	Convenience	Medical students	635, 66.9% female, 20.6 (1.8) mean age	Intention to specialize abroad	73.7
Tabib et al., 2022 [58]	Tunisia (LM, No)	Convenience	Interns and residents	56, NR female, NR mean age	Plans to immigrate and work abroad	46.4

^1^Total sample size (n), percentage of female participants (%), and mean age with standard deviation (SD), unless otherwise specified. FCAS: Fragile and Conflict-Affected Situations; H: High-income; L: Low-income; LM: Lower-middle-income; NA: Not applicable; NR: Not reported; UM: Upper-middle-income

All included studies employed a cross-sectional design and were published between 2008 and 2026. Most studies employed non-probabilistic convenience sampling (*n* = 19), while five studies did not report their sampling technique. All studies were conducted within a single country across 10 Arab countries, except for one multi-center study [46]. Egypt was the most frequently represented country (*n* = 8), followed by Sudan (*n* = 5). Based on World Bank income classifications, more than half of the included studies originated from LMIC economies (*n* = 18), and a substantial proportion (*n* = 14) was conducted in FCAS countries, specifically Sudan, Palestine, Iraq, and Syria.

The pooled percentage of female participants was approximately 58%, and the mean age of participants clustered around 21 years (ranging from 20.6 to 27.7 years). The majority of the studies (*n* = 22) surveyed medical students, either as the exclusive focus or in combination with interns and residents.

### Risk of Bias Assessment of Included Studies

Regarding study sample selection, 28 studies were considered somewhat representative of the target population based on non-random sampling, while three studies did not provide sufficient information to describe their sampling strategy. Sample size was considered justified and satisfactory in 16 studies, whereas 15 studies did not provide a satisfactory justification for their sample size. For outcome assessment, 16 studies used an acceptable assessment tool, while 15 studies did not adequately describe the assessment tool used. Detailed assessments for each included study are presented in Table S2 (Additional file 1).

### Operational Definitions of Migration Intention

Across the 31 included studies, the operational definitions of migration intention varied and were classified into three primary domains. The most frequently assessed outcome was training-related migration (*n* = 13), with definitions within this category including “travel for specialty or subspecialty training” [34], “preference for pursuing residency abroad” [36], and “interested in specializing abroad” [54]. A nearly equal proportion of studies evaluated general migration intentions (*n* = 12), utilizing broad definitions such as “plan to emigrate either alone or with family” [22], “wish to migrate” [43], and “intentions to leave country permanently” [15]. The remaining studies measured work-related migration (*n* = 6), which specifically targeted long-term occupational placement using definitions like “preferred future work location” [32], “plan on pursuing their career abroad” [33], and “plans to immigrate and work abroad” [58].

### Prevalence of Migration Intention

#### Overall Prevalence of Migration Intention

**Fig. 2:**
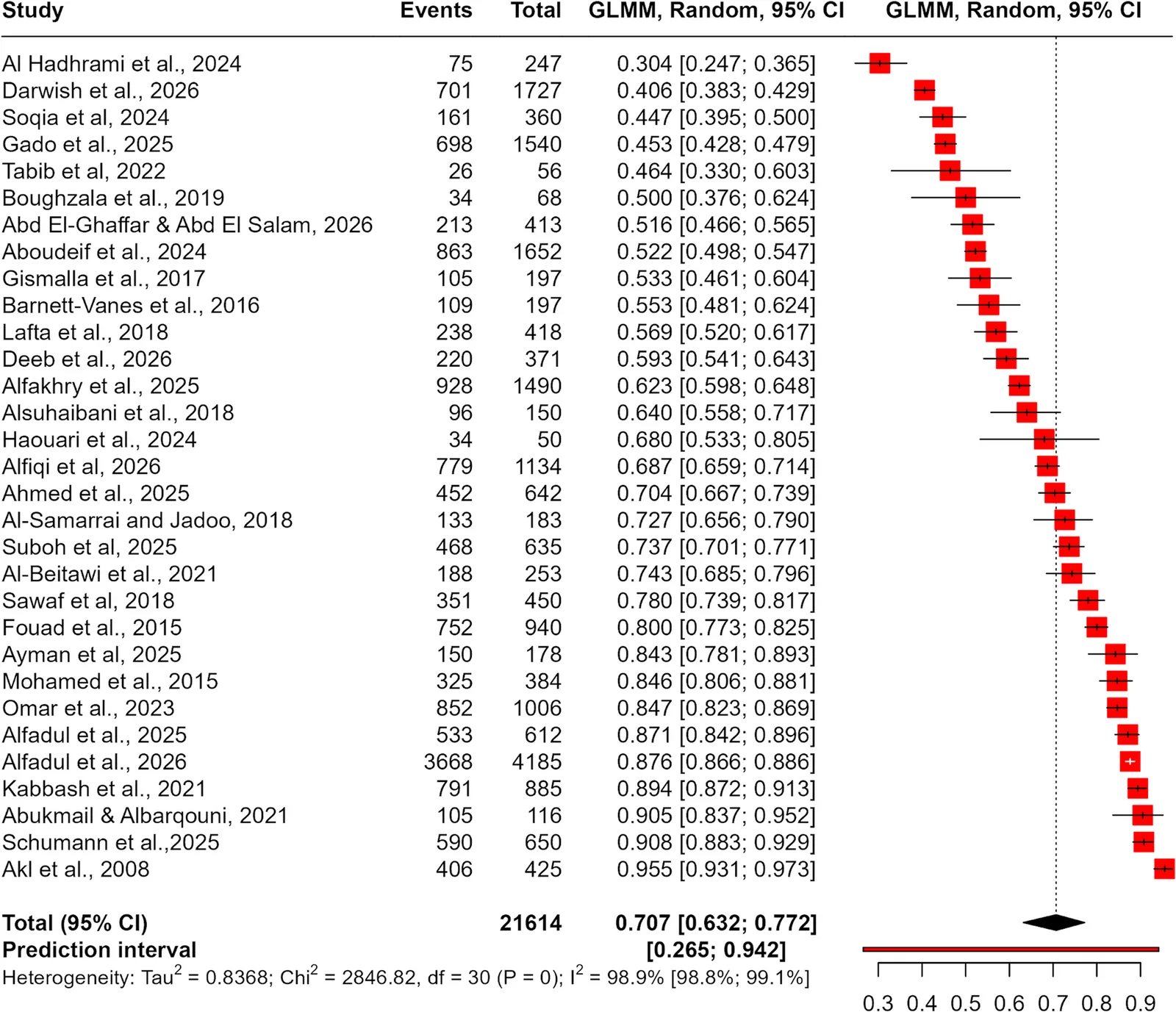
Forest plot of the prevalence of migration intention among Arab medical trainees

The meta-analysis included 31 studies comprising a total of 21,614 participants and 15,044 events. Using a random-effects model, the overall pooled prevalence of migration intention was 70.7% (95% CI: 63.2%, 77.2%). A high degree of absolute heterogeneity was observed across the included studies (Tau² = 0.84). The 95% prediction interval ranged from 26.5% to 94.2%, indicating substantial variability in true prevalence across different settings (Fig. 2).

#### Subgroup Analyses

**Fig. 3:**
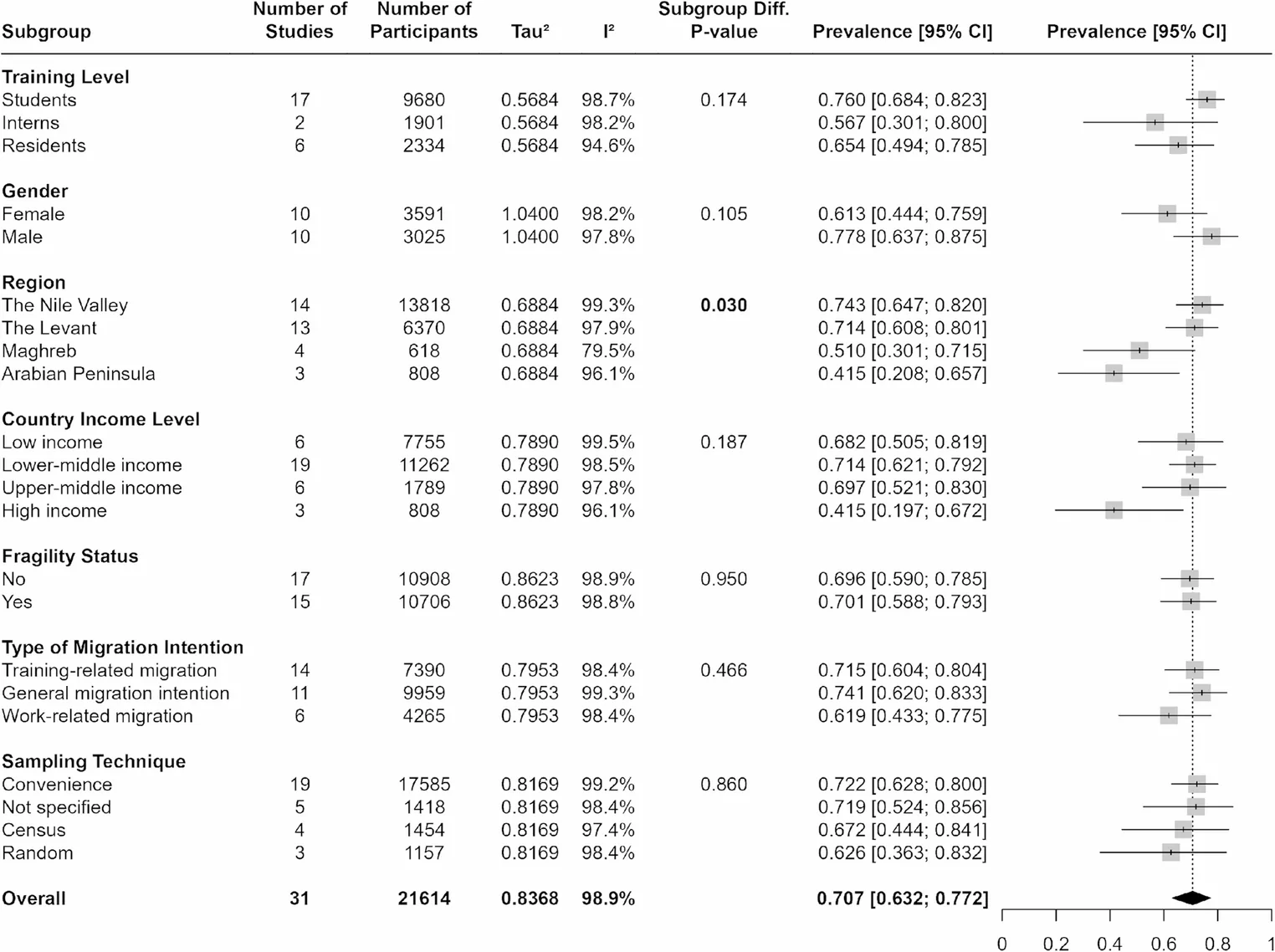
Summary forest plot of subgroup analyses for the prevalence of migration intention among Arab medical trainees

Subgroup analyses were conducted based on training level, gender, region, country income level, fragility status, type of migration intention, and sampling technique (summarized in Fig. 3).

The pooled prevalence was highest among students at 76.0% (95% CI: 68.4%, 82.3%) across 17 studies, followed by residents at 65.4% (95% CI: 49.4%, 78.5%) across 6 studies, and interns at 56.7% (95% CI: 30.1%, 80.0%) across 2 studies, although the differences were not statistically significant (*p* = 0.174; Figure S1; Additional file 1). Notably, however, subgrouping by training level resulted in the largest decrease in heterogeneity, lowering the residual Tau² to 0.57 (compared to the overall Tau² of 0.84). On the other hand, pooled prevalence was notably higher among male trainees (77.8%, 95% CI: 63.7%, 87.5%) compared to female trainees (61.3%, 95% CI: 44.4%, 75.9%) although the differences were also not statistically significant (*p* = 0.105; Figure S2; Additional file 1).

Regional differences were statistically significant (*p* = 0.030), and this subgrouping also notably reduced the residual Tau² to 0.69. Studies from The Nile Valley reported the highest prevalence at 74.3% (95% CI: 64.7%, 82.0%) across 14 studies, closely followed by The Levant at 71.4% (95% CI: 60.8%, 80.1%) across 13 studies. In comparison, Maghreb and Arabian Peninsula reported much lower estimates at 51.0% (95% CI: 30.1%, 71.5%) and 41.5% (95% CI: 20.8%, 65.7%), respectively (Figure S3; Additional file 1). This is further reflected in the subgroup analysis by country income, where LMICs reported the highest prevalence (71.4%, 95% CI: 62.1%, 79.2%), while high-income countries, namely the Arabian Peninsula studies, reported the lowest (41.5%, 95% CI: 19.7%, 67.2%) (Figure S4; Additional file 1).

Notably, fragility status showed no statistically significant difference (*p* = 0.950), with nearly comparable migration intention prevalences of 70.1% (95% CI: 58.8%, 79.3%) in fragile settings compared to 69.6% (95% CI: 59.0%, 78.5%) in non-fragile settings (Figure S5; Additional file 1).

General migration intention had the highest prevalence (74.1%, 95% CI: 62.0%, 83.3%), followed by training-related migration (71.5%, 95% CI: 60.4%, 80.4%) and work-related migration (61.9%, 95% CI: 43.3%, 77.5%) (Figure S6; Additional file 1).

Although sampling technique did not yield statistically significant differences (*p* = 0.860), the pooled prevalence of migration intention in studies utilizing random sampling (62.6%, 95% CI: 36.3%, 83.2%) was nearly 10% less than that among studies that utilized convenience sampling (72.2%, 95% CI: 62.8%, 80.0%) (Figure S7; Additional file 1).

#### Sensitivity Analysis

A leave-one-out sensitivity analysis was performed to determine if any single study heavily influenced the overall pooled prevalence. The omission of any individual study yielded pooled prevalence estimates ranging between 69.2% and 71.9%, indicating that the overall estimate was relatively consistent. However, the omission of the study by Akl et al. [16] resulted in the largest decrease in heterogeneity, reducing Tau² to 0.71 compared to the overall Tau² of 0.84 (Figure S8; Additional file 1).

### Factors Influencing Migration Intentions

Drivers of migration intention were reported in 19 studies. The most commonly reported push factors driving individuals away included ongoing war and insecurity (k = 10) [22, 35, 39, 48, 51–55, 58], low pay and financial insecurity (k = 9) [14, 15, 43, 45, 48, 51, 54, 55, 58], and poor working conditions (k = 7) [14, 43, 45, 48, 51, 56, 58]. Other push factors included workplace violence and abuse [14, 41, 43, 45, 51], low social status and poor public image [14, 34, 43, 45, 48], and poor training and research opportunities [14, 15, 45, 48, 51].

Conversely, pull factors attracting individuals abroad were predominantly professional and economic, including higher pay and financial security (k = 11) [15, 22, 35, 41, 45, 48, 50, 52–54, 56], better training and research opportunities (k = 10) [14, 15, 35, 41, 45, 48, 50, 52, 54, 56], and better working conditions and career growth (k = 10) [15, 35, 41, 45, 48, 50, 52–55]. Other pull factors included a desire for a better quality of life [15, 22, 35, 41, 45, 48, 52, 56], political stability and safety [22, 41, 48, 56], and social networks and prestige abroad [41, 48, 50, 52].

Mooring factors anchoring individuals to their home country included strong family ties and a sense of belonging (k = 6) [15, 35, 48, 50, 52, 56], a sense of patriotism and duty to the country (k = 5) [35, 48, 50, 52, 56], and cultural and religious ties (k = 3) [16, 48, 54]. Conversely, mooring factors that facilitated their migration included existing diaspora networks abroad [15, 50, 54, 55, 58], gender and dual nationality [15, 37, 47, 54, 58] and the availability of visas, exams and travel costs [15, 22, 54]. All reported factors are included in Table S3 in Additional file 1, with a descriptive thematic summary shown in Table 2.

**Table 2:** Push, pull, and mooring factors influencing Arab trainees’ migration intentions

Category	Thematic Factor	Reporting studies (*n*)
Push Factors	War, Conflict & Insecurity [22, 35, 39, 48, 51–55, 58]	10
	Low Pay & Financial Insecurity [14, 15, 43, 45, 48, 51, 54, 55, 58]	9
	Poor Working Conditions [14, 43, 45, 48, 51, 56, 58]	7
	Workplace Violence & Abuse [14, 41, 43, 45, 48, 51]	6
	Poor Training & Research Opportunities [14, 15, 45, 48, 51]	5
	Low Social Status & Poor Public Image [14, 34, 43, 45, 48]	5
Pull Factors	Higher Pay & Financial Security [15, 22, 35, 41, 45, 48, 50, 52–54, 56]	11
	Better Training & Research Opportunities [14, 15, 35, 41, 45, 48, 50, 52, 54, 56]	10
	Better Working Conditions & Career Growth [15, 35, 41, 45, 48, 50, 52–55]	10
	Better Quality of Life [15, 22, 35, 41, 45, 48, 52, 56]	8
	Political Stability & Safety [22, 41, 48, 56]	4
	Social Networks & Prestige Abroad [41, 48, 50, 52]	4
Mooring Factors (Anchors)	Family Ties & Sense of Belonging [15, 35, 48, 50, 52, 56]	6
	Patriotism & Duty to Country [35, 48, 50, 52, 56]	5
	Cultural & Religious Ties [16, 48, 54]	3
Mooring Factors (Facilitators)	Diaspora Networks Abroad [15, 50, 54, 55, 58]	5
	Male Gender & Dual Nationality [15, 37, 47, 54, 58]	5
	Visa, Exams & Travel Costs [15, 22, 54]	3

## Discussion

Placed globally, our pooled estimate of 70.7% falls toward the upper end threshold of the 20.8% to 89.6% range reported by Ser et al. [59] in their scoping review of international migration trends among medical students. Notably, the countries with high migration intentions in that review spanned both low- and high-income contexts, suggesting that a country’s macroeconomic status alone does not entirely dictate migration behaviors. This aligns with our subgroup analyses, which demonstrated consistently high intention rates (68.2% to 71.4%) across low-, lower-middle-, and upper-middle-income Arab nations. In contrast, the lowest prevalence of migration intent (41.5%) was observed only among HICs of the Arabian Peninsula, where well-resourced domestic health systems likely function as regional destinations rather than sources of out-migration. In line with this, evidence shows that both brain drain/gain and brain waste are predominantly observed in migration flows from LICs or LMICs to HICs [60]. This pattern is further supported by our thematic synthesis findings, in which advanced training and academic opportunities, along with improved working conditions and prospects for professional advancement, emerged as the most frequently reported pull factors. Furthermore, the exceptionally wide 95% prediction interval (26.5% to 94.2%) indicates substantial variation in migration intentions across different settings. Consequently, the overall pooled prevalence of 70.7% should not be assumed as a universal baseline for all Arab countries. From a practical standpoint, this interval highlights that local health workforce planning must be heavily contextualized.

Nevertheless, a notable finding in our review is the absence of a significant difference in migration intentions between trainees in FCAS and non-FCAS settings (70.1% vs. 69.6%). While armed conflict may be assumed to be a dominant driver of emigration, this pattern suggests that structural and economic constraints in ostensibly stable contexts may exert a comparably strong push. This interpretation is consistent with the systematic review of 107 studies on drivers of health workers’ migration intention and non-migration from low- and middle-income countries, which identified poor remuneration and limited career prospects as consistent drivers of migration across settings, irrespective of conflict status [61]. Furthermore, the lack of elevated migration intent among trainees in FCAS settings may be influenced by the survey administration timing and feasibility of migration within the conflict setting. As suggested by longitudinal OECD data, physician emigration typically peaks prior to conflict onset and subsequently declines [9]. Therefore, individuals with the greatest capability and intention to migrate may have already left prior to conflict escalation and survey administration, while those remaining in FCAS faced substantial logistical and administrative barriers that constrained international mobility and may have reduced migration intentions. This pattern was reflected in two included studies. In Iraq, 62% of medical students reported frequent severe security threats that necessitated immediate internal displacement and created major obstacles to migration abroad [44]. Similarly, in a study among Syrian students, difficulty obtaining a visa was the most commonly reported barrier to migration, reported by more than half of the study’s participants [54]. Together, these factors may have contributed to the comparable levels of reported migration intention observed across FCAS and non-FCAS settings; however, these interpretations remain speculative and should be considered in light of the limitations of the available data.

We also found that migration intentions were highest among undergraduates (76.0%) and lower among interns (56.7%) and residents (65.4%), although the differences were not significant, and the estimate for interns should be interpreted cautiously given the limited number of studies (k = 2). This decline may reflect increasing exposure to structural barriers to migration across the training continuum such as credentialing requirements, visa constraints, and the competitiveness of international residency pathways, prompting more pragmatic reassessment of migration feasibility. In parallel, cumulative investment in local training and social anchoring (e.g., marriage) may further discourage migration at later stages, consistent with our finding that family and social ties were the most commonly reported mooring factors. A similar pattern may help explain the lower migration intention among female trainees compared to males (61.3% vs. 77.8%). This difference may reflect gendered socio-cultural and logistical constraints on mobility in the Arab region, including family expectations and caregiving obligations, rather than greater satisfaction with domestic systems [62, 63]. Together, these factors may contribute to the observed variations by training stage and sex. However, because these subgroup comparisons are based on study-level observational data, differences may be subject to unmeasured confounding factors and should be interpreted as exploratory hypotheses rather than independent causal effects, especially for subgroups with limited study counts.

Finally, our descriptive synthesis utilizing the PPM framework aligns closely with the global determinants of medical students’ migration intention recently outlined by Ser et al. [59]. In both the Arab region and the global context, trainee exodus appears to be overwhelmingly driven by structural push factors such as financial instability, subpar working conditions, and geopolitical conflict. Conversely, the primary pull of destination countries remains anchored in the promise of advanced academic training and financial prosperity. While personal mooring factors such as strong family ties and a patriotic duty to the community act as friction against migration, our findings suggest they are largely insufficient to counterbalance severe systemic deficiencies. Consequently, to meaningfully mitigate workforce attrition, our findings suggest that regional health ministries should transition toward tangible structural reforms, prioritizing the expansion of robust domestic residency pathways, ensuring occupational safety against workplace violence, and establishing competitive, inflation-adjusted remuneration models. Nevertheless, our findings, including those related to PPM factors, pertain to migration intentions rather than actual migration behavior. As intentions do not necessarily translate into future migration, these findings should therefore be interpreted with caution.

### Limitations

This systematic review and meta-analysis has several limitations. Substantial statistical heterogeneity was observed across pooled estimates, likely reflecting the multifactorial nature of migration intentions and unmeasured contextual differences between countries, institutions, and survey periods that could not be fully accounted for using study-level data. This high degree of unmeasured contextual variation is mathematically reflected in the wide prediction interval, underscoring that the summary estimate of 70.7% cannot be reliably extrapolated to any single unrepresented training context or individual country. In addition, there is currently no standardized definition or validated instrument for assessing physician migration intentions, and the included studies used varying operational definitions of migration intent, resulting in substantial remaining heterogeneity in the pooled prevalence estimates, and weakening the strength of the results. All included studies were cross-sectional and based on self-reported survey data, making the findings susceptible to sampling and reporting biases. The geographical distribution of studies was also uneven, which may limit the regional generalizability of the pooled estimates. We also included preprints, which although are unlikely to differ in their reported prevalence estimates post-review, may nonetheless weaken the certainty of the results. Further, although potentially overlapping datasets were identified and excluded based on similarities in participant and methodological characteristics, we could not determine with complete certainty whether individual participants were included in more than one study when sufficient information was not available. Therefore, some degree of participant overlap and consequent double counting cannot be completely excluded.

Lastly, we did not formally assess publication bias. As Borenstein argues, publication bias is conceptually misaligned with prevalence meta-analyses because these studies estimate epidemiological proportions rather than comparative effect sizes [64]. Consequently, there is no analogous “positive” or “negative” result that would be expected to influence publication in the same manner as intervention or association studies. Furthermore, conventional methods such as funnel plots and Egger’s regression are based on assumptions that are frequently violated in meta-analyses of proportions, as prevalence estimates are bounded between 0 and 100% and their standard errors are intrinsically related to the observed prevalence, which can produce funnel plot asymmetry even in the absence of publication bias. Therefore, applying standard publication bias procedures to prevalence meta-analyses may yield misleading results and is generally not recommended [65].

## Conclusion and Policy Implications

This systematic review and meta-analysis found that more than two-thirds of medical trainees in the Arab region intended to migrate, highlighting a substantial health workforce challenge. Our descriptive synthesis PPM findings suggest that improving physician retention will likely require structural interventions, including competitive salaries, safer working environments, and stronger local postgraduate training opportunities. From a health workforce planning perspective, these findings highlight the scale of migration intent and suggest potential priority areas for retention policies, including professional development, workplace safety, and remuneration.

## Supplementary Information

Below is the link to the electronic supplementary material.

Supplementary Material 1

## Data Availability

The dataset utilized in this study is available from the corresponding author upon reasonable request.

## References

[1] Sweileh W. Research Trends and Patterns on International Migration of Health Workers (1950–2022). Sage Open. 2024;14:21582440241293190. 10.1177/21582440241293190.

[2] Aluttis C, Bishaw T, Frank MW. The workforce for health in a globalized context – global shortages and international migration. Glob Health Action. 2014;7:23611. 10.3402/gha.v7.23611. PMC3926986 24560265

[3] International migration of health professionals to OECD countries: International Migration Outlook. 2025. OECD. 2025. https://www.oecd.org/en/publications/international-migration-outlook-2025_ae26c893-en/full-report/international-migration-of-health-professionals-to-oecd-countries_fea88ae4.html. Accessed 30 Apr 2026.

[4] Misau YA, Al-Sadat N, Gerei AB. Brain-drain and health care delivery in developing countries. J Public Health Afr. 2010;1:2. 10.4081/jphia.2010.e6. PMC5345397 28299040

[5] Botezat A, Ramos R. Physicians’ brain drain - a gravity model of migration flows. Glob Health. 2020;16:7. 10.1186/s12992-019-0536-0. PMC6961279 31937356

[6] Lofters A, Slater M, Fumakia N, Thulien N. Brain drain and brain waste: experiences of international medical graduates in Ontario. Risk Manag Healthc Policy. 2014;7:81–9. 10.2147/RMHP.S60708. PMC4027850 24868176

[7] Chen C, Bernard A, Rylee R, Abel G. Brain Circulation: The Educational Profile of Return Migrants. Popul Res Policy Rev. 2022;41:387–99. 10.1007/s11113-021-09655-6. PMC8075014 33935340

[8] World Bank Group. Classification of Fragile and Conflict-Affected Situations. World Bank; 2025. https://www.worldbank.org/en/topic/fragilityconflictviolence/brief/classification-of-fragile-and-conflict-affected-situations. Accessed 24 Apr 2026.

[9] Ramesh T, Wozniak GD, Horvitz-Lennon M, Zhang F, Yu H. Trends in Physician Emigration from Conflict-Affected Countries, 2006–2021. J Gen Intern Med. 2026;41:881–4. 10.1007/s11606-025-09791-1. PMC12960881 40762876

[10] World Health Organization Regional Office for the Eastern Mediterranean. Health and well-being profile of the Eastern Mediterranean Region: an overview of the health situation in the Region and its countries in 2023. Cairo: WHO; 2024.

[11] Kronfol NM. Historical development of health professions’ education in the Arab world. East Mediterr Health J. 2012;18(11):1157–65. 10.26719/2012.18.11.1157. 23301379

[12] Tekian A, Boulet J. A longitudinal study of the characteristics and performances of medical students and graduates from the Arab countries. BMC Med Educ. 2015;15:200. 10.1186/s12909-015-0482-3. PMC4634602 26537506

[13] Admin WHO. May. Health workforce in the Eastern Mediterranean Region: from COVID-19 lessons to action. https://www.emro.who.int/media/news/health-workforce-in-the-eastern-mediterranean-region-from-covid-19-lessons-to-action.html. Accessed 1 2026. 10.1002/hpm.3756 38163282

[14] Kabbash I, El-Sallamy R, Zayed H, Alkhyate I, Omar A, Abdo S. The brain drain: why medical students and young physicians want to leave Egypt. East Mediterr Health J. 2021;27:1102–8. 10.26719/emhj.21.050. 34927714

[15] Omar Y, Al Refaei A, Al-Qawasmeh AR, Ghazzal H, Kalbouneh H. Migration intent among Jordanian medical students: a nationwide cross-sectional investigation. Postgrad Med J. 2023;99:855–61. 10.1136/postgradmedj-2022-141817. 37137543

[16] Akl EA, Maroun N, Major S, Afif C, Abdo A, Choucair J, et al. Post-graduation migration intentions of students of Lebanese medical schools: a survey study. BMC Public Health. 2008;8:191. 10.1186/1471-2458-8-191. PMC2424042 18518954

[17] Tjaden J, Auer D, Laczko F. Linking Migration Intentions with Flows: Evidence and Potential Use. Int Migr. 2019;57:36–57. 10.1111/imig.12502.

[18] Walton-Roberts M, Bourgeault IL. Health workforce data needed to minimize inequities associated with health-worker migration. Bull World Health Organ. 2024;102:117–22. 10.2471/BLT.23.290028. PMC10835628 38313146

[19] Krishnan G, Raghuram JNV. Exploring factors and contextual applications of the Push-Pull Mooring (PPM) framework in switching intention: A systematic literature review. Multidiscip Rev. 2024;7:2024003–2024003. 10.31893/multirev.2024003.

[20] Nemr E, Moussallem M, Nemr R, Kosremelli Asmar M. Exodus of Lebanese doctors in times of crisis: a qualitative study. Front Health Serv. 2023;3. 10.3389/frhs.2023.1240052. PMC10643131 38028945

[21] Almansour H, Aldossary A, Holmes S, Alderaan T. Migration of nurses and doctors: pull factors to work in Saudi Arabia. Hum Resour Health. 2023;21:25. 10.1186/s12960-023-00809-5. PMC10029214 36941641

[22] Al-Samarrai MAM, Ali Jadoo SA. Iraqi medical students are still planning to leave after graduation. J Ideas Health. 2018;1:23–8. 10.47108/jidhealth.Vol1.Iss1.5.

[23] Sheikh JI, Cheema S, Chaabna K, Lowenfels AB, Mamtani R. Capacity building in health care professions within the Gulf cooperation council countries: paving the way forward. BMC Med Educ. 2019;19:83. 10.1186/s12909-019-1513-2. PMC6417223 30871521

[24] Page MJ, McKenzie JE, Bossuyt PM, Boutron I, Hoffmann TC, Mulrow CD, et al. The PRISMA 2020 statement: an updated guideline for reporting systematic reviews. BMJ. 2021;372:n71. 10.1136/bmj.n71. PMC8005924 33782057

[25] Carling J. How Does Migration Arise? Geneva: IOM; 2017.

[26] Balduzzi S, Rücker G, Schwarzer G. How to perform a meta-analysis with R: a practical tutorial. Evid Based Ment Health. 2019;153–60. 10.1136/ebmental-2019-300117. PMC10231495 31563865

[27] World Bank Group. World Bank Country and Lending Groups. World Bank. 2026. https://datahelpdesk.worldbank.org/knowledgebase/articles/906519-world-bank-country-and-lending-groups. Accessed 24 Apr 2026.

[28] Carra MC, Romandini P, Romandini M. Risk of Bias Evaluation of Cross-Sectional Studies: Adaptation of the Newcastle‐Ottawa Scale. J Periodontal Res. 2025. 10.1111/jre.13405. PMC13665615 40293188

[29] R Core Team. R: A Language and Environment for Statistical Computing. Vienna, Austria: R Foundation for Statistical Computing; 2025.

[30] Viechtbauer W. Conducting meta-analyses in R with the metafor package. J Stat Softw. 2010;36:1–48. 10.18637/jss.v036.i03.

[31] Rücker G, Schwarzer G, Carpenter JR, Schumacher M. Undue reliance on I(2) in assessing heterogeneity may mislead. BMC Med Res Methodol. 2008;8:79. 10.1186/1471-2288-8-79. PMC2648991 19036172

[32] Abd El-Ghaffar YS, Abd El Salam A. Specialty preferences and influencing factors among undergraduate medical students in Ain Shams University. BMC Med Educ. 2026;26:335. 10.1186/s12909-026-08603-2. PMC12930952 41668112

[33] Aboudeif A, Elaraby Y, Hany M, Nasser S, Refaat N, Mohamed YG, et al. Inherited privilege? First vs. continuing-generation medical students in Egypt, academic performance, extracurricular training and expectations: a cross-sectional study. BMC Med Educ. 2024;24:1274. 10.1186/s12909-024-06227-y. PMC11542418 39506796

[34] Abukmail E, Albarqouni L. Postgraduate training abroad and migration intentions of medical doctors and students in Gaza: a cross-sectional survey. Lancet. 2021;398:S6. 10.1016/S0140-6736(21)01492-6. PMC8617349 34227988

[35] Ahmed SMM, Masaad MB, Abdulrahman MSA, Mohamedzain EMI. Challenges and Choices Amidst Military Conflict: The Present and Future of Medical Education and Healthcare in Sudan. Health Sci Rep. 2025;8:e70334. 10.1002/hsr2.70334. PMC11711230 39790215

[36] Said Al Hadhrami R, Khalfan Al Mamari AS, Hamed Al Omrani S, Hummod Al Rumaidhi S, Mohammed Al Busaidi T, Mohamed Al Kindi R, et al. Factors predicting the choice of specialization among medical students and junior doctors in Oman: a cross-sectional study. J Med Educ. 2024;23. 10.5812/jme-145508.

[37] Al-beitawi SN, Al-Shatanawi TN, Qudsieh SA, Abu Marar EI, Al Zoubi MS, Al-zubi M. 6th year medical students’ future specialty preferences: A cross-sectional study. Ann Med Surg. 2021;66. 10.1016/j.amsu.2021.102373. PMC8141496 34040768

[38] Alfadul ESA, Alrawa SSK, Hemmeda L, Adam AYT, Mohamed Salih Mohamed Nour S, Ebrahim Hamed Saeed N, et al. Effect of military conflict on mental health: a cross-sectional study among the medical students at Khartoum governmental universities, Sudan, 2023. BMJ Open. 2025;15:e086495. 10.1136/bmjopen-2024-086495. PMC11956355 40132815

[39] Alfadul ESA, Alrawa SS, Tebaig B, Molodynski A, Osman AHM. Mental health disorders among Sudanese medical students amid the 2023 conflict: prevalence, predictors, and implications. BMC Psychiatry. 2026;26:217. 10.1186/s12888-026-07843-2. PMC12947355 41620672

[40] Alfakhry G, Kodmani R, Ghandour M, Al Munajjed A, Khwanda R. Restoring the Clinical Learning Environment at Teaching Hospitals in Post-Assad Syria: A PHEEM Analysis and Recommendations. MedEdPublish. 2025;15:15. 10.12688/mep.20955.2. PMC12082068 40386493

[41] Alfiqi R, Elkholi MA, Azzam H, et al. Emigration intentions and their association with workplace violence among Egyptian medical students and early career physicians. Discov Public Health. 2026;23:989. 10.1186/s12982-026-02330-1.

[42] Alsuhaibani M, Alharbi A, Alqaryan SK, Aldress T, Alharbi M, Alharethy S. The impact of the Brain Drain involving Saudi Physicians: A Cross-Sectional Study. J Nat Sci Med. 2018;1:69. 10.4103/JNSM.JNSM_32_18.

[43] Ayman A, Negm AY, Raheem A, Awadallah M, Ismail A, Baz M et al. Why are they escaping? Factors influencing medical student migration intentions in Egypt: a cross-sectional study. 2025. 10.21203/rs.3.rs-6561892/v1

[44] Barnett-Vanes A, Hassounah S, Shawki M, Ismail OA, Fung C, Kedia T, et al. Impact of conflict on medical education: a cross-sectional survey of students and institutions in Iraq. BMJ Open. 2016;6:e010460. 10.1136/bmjopen-2015-010460. PMC4762136 26883241

[45] Boughzala W, Hariz A, Badri T, Ben Hassine L, Azzabi S, Khalfallah N. Family medicine: attractiveness, constraints and prospects as perceived by residents of the specialty. Tunis Med. 2019;97:904–9. 31872402

[46] Darwish NE, Omar YM, Elsaadany MA, Darwish Y, Johar O, Alhussein H et al. Arab medical students’ attitudes toward the arabization of medical education: a multiregional cross-sectional study. 2026. 10.21203/rs.3.rs-9349031/v1 PMC13373500 42465710

[47] Deeb N, Deeb S, Amro AM, Alhashlamon K, Abuzahra A, Hamad S, et al. Clinical training under movement restriction: checkpoint burden, burnout, and intentions to train abroad among medical students in Palestine. BMC Med Educ. 2026;26:698. 10.1186/s12909-026-09049-2. PMC13130462 41864907

[48] Fouad YA, Fahmy YM, Abdel Hady SM, Elsabagh AE. Egyptian future physicians are packing to leave but may be willing to return. Int Health. 2015;7:190–4. 10.1093/inthealth/ihu072. 25256738

[49] Gado MI, Seliem SM, Ali AM, Alqedra JJF, Elbadry DF, El-Gilany AH. Nationwide cross-sectional study of factors affecting specialty choice among medical interns in Egypt. Res Square [Preprint]. 2025. 10.21203/rs.3.rs-7402329/v1.

[50] Gismalla M, Abdallah A, Mohammed MA, Mahjoub Taha S. Factors influence abroad or home post graduates training among medical students: experience from Sudan. Int J Med. 2017;5:109–12. 10.14419/ijm.v5i1.7439.

[51] Haouari W, Omri S, Labyadh A, Feki R, Gassara I, Charfi N, et al. Factors Driving the Emigration Intentions of Young Tunisian Medical Professionals. Eur Psychiatry. 2024;67:S829–30. 10.1192/j.eurpsy.2024.1731.

[52] Lafta R, Al-Ani W, Dhiaa S, Cherewick M, Hagopian A, Burnham G. Perceptions, experiences and expectations of Iraqi medical students. BMC Med Educ. 2018;18:53. 10.1186/s12909-018-1156-8. PMC5870482 29587726

[53] Mohamed E, Balla S, Abdalla A, Yousif M, Alzahrani M, Medani K, et al. Post-graduation migration intentions of medical students in the Faculty of Medicine, University of Khartoum. World J Pharm Pharm Sci. 2015;4:26–33.

[54] Sawaf B, Abbas F, Idris A, Al Saadi T, Ibrahim N. Specialty preference and intentions to study abroad of Syrian medical students during the crisis. BMC Med Educ. 2018;18:39. 10.1186/s12909-018-1146-x. PMC5857144 29548290

[55] Schumann M, Elsayed Mohamed Amer M, Abdelhady Mashaal NM, Abd El-Hamed Montasser N, Aref Mostafa SR. Factors Influencing Migration Intentions of Undergraduate Medical Students: a cross-sectional study from two Egyptian universities. J Health Prof Educ Innov. 2025;2:28–46. 10.21608/jhpei.2025.344651.1038.

[56] Soqia J, Yakoub-Agha L, Mohamad L, Alhomsi R, Shamaa MA, Yazbek A, et al. Syrian crises effect on specialty choice and the decision to work in the country among residents of six major hospitals in Syria, Damascus. PLoS ONE. 2024;19:e0295310. 10.1371/journal.pone.0295310. PMC10852239 38329984

[57] Suboh MW, Atatre MH, Dababseh BH, Ghnimat AS, AbuHamdiya LW, Alhaddad MS. Motivations, satisfaction, and career aspirations of undergraduate medical students in West Bank, Palestine: a cross-sectional study. BMC Med Educ. 2025;25:1637. 10.1186/s12909-025-08230-3. PMC12642258 41286843

[58] Tabib F, Guermazi F, Zouari A, Ben Abdallah M, Hentati S, Baati I, et al. Emigration intentionality among Tunisian interns and residents in medicine. Eur Psychiatry. 2022;65(Suppl 1):S217. 10.1192/j.eurpsy.2022.564.

[59] Ser GTZ, Teng ZS, Yusni YZ, Ooi KSY, Wong ZY, Tejpal Singh PS, et al. A scoping review on medical students’ international migration: trends, determinants, and implications for global health workforce planning. Hum Resour Health. 2025;23:67. 10.1186/s12960-025-01027-x. PMC12642250 41287130

[60] Adovor E, Czaika M, Docquier F, Moullan Y. Medical brain drain: How many, where and why? J Health Econ. 2021;76:102409. 10.1016/j.jhealeco.2020.102409. 33465558

[61] Toyin-Thomas P, Ikhurionan P, Omoyibo EE, Iwegim C, Ukueku AO, Okpere J, et al. Drivers of health workers’ migration, intention to migrate and non-migration from low/middle-income countries, 1970–2022: a systematic review. BMJ Glob Health. 2023;8:e012338. 10.1136/bmjgh-2023-012338. PMC10174016 37156560

[62] McLean M, Higgins-Opitz SB. Male and Female Emirati Medical Clerks’ Perceptions of the Impact of Gender and Mobility on Their Professional Careers. Soc Sci. 2017;6:109. 10.3390/socsci6030109.

[63] Dar-Odeh N, Elsayed SA, Nourwali I, Ryalat S, Al-Shayyab MH, Abu-Hammad O. Social factors as career obstacles for female oral and maxillofacial surgeons in three Middle Eastern countries. Int J Oral Maxillofac Surg. 2019;48:1504–8. 10.1016/j.ijom.2019.03.001. 30910411

[64] Borenstein M, Hedges LV, Higgins JPT, Rothstein HR. Introduction to Meta-Analysis. 2nd ed. Hoboken, NJ Chichester: Wiley; 2021.

[65] Barendregt JJ, Doi SA, Lee YY, Norman RE, Vos T. Meta-analysis of prevalence. J Epidemiol Community Health. 2013;67(11):974–8. 10.1136/jech-2013-203104. 23963506

