# Supplementary material for: Pushed, Pulled, and Moored: A Systematic Review and Meta-analysis of Migration Intentions Among Medical Trainees in the Arab Region
Source: J Epidemiol Glob Health. 2026 Oct 2;16(1):123. doi: 10.1007/s44197-026-00640-w (PMC13633222; doi:10.1007/s44197-026-00640-w)
Supplement: Supplementary file 1 [file 44197_2026_640_MOESM1_ESM.docx]

**Table S1.** Search Strategy.

| **Database** | **Search strategy** | **Results** |
| --- | --- | --- |
| **PubMed** | ("Students, Medical"[Mesh] OR "Internship and Residency"[Mesh] OR "medical student*"[tiab] OR "medical undergraduate*"[tiab] OR "undergraduate medicine"[tiab] OR "medical intern*"[tiab] OR "intern doctor*"[tiab] OR "intern physician*"[tiab] OR "medical resident*"[tiab] OR "resident doctor*"[tiab] OR "resident physician*"[tiab]) AND ("Emigration and Immigration"[Mesh] OR "Career Choice"[Mesh] OR migrat*[tiab] OR emigrat*[tiab] OR immigrat*[tiab] OR "brain drain"[tiab] OR "working abroad"[tiab] OR "work abroad"[tiab] OR "studying abroad"[tiab] OR "study abroad"[tiab] OR "training abroad"[tiab] OR "train abroad"[tiab] OR "intention to leave"[tiab] OR "stay intention*"[tiab] OR "career intention*"[tiab] OR "future career"[tiab] OR workforce[tiab] OR "leaving the country"[tiab] OR "work overseas"[tiab]) AND ("Arab Countries"[Mesh] OR "Africa, Northern"[Mesh] OR "Middle East"[Mesh] OR Arab*[tiab] OR "Middle East"[tiab] OR "North Africa"[tiab] OR MENA[tiab] OR Algeria*[tiab] OR Bahrain*[tiab] OR Comoros[tiab] OR Djibouti[tiab] OR Egypt*[tiab] OR Iraq*[tiab] OR Jordan*[tiab] OR Kuwait*[tiab] OR Lebanon[tiab] OR Lebanese[tiab] OR Libya*[tiab] OR Mauritania*[tiab] OR Morocco[tiab] OR Moroccan[tiab] OR Oman*[tiab] OR Palestin*[tiab] OR Qatar*[tiab] OR "Saudi Arabia"[tiab] OR Saudi[tiab] OR KSA[tiab] OR Somalia*[tiab] OR Sudan*[tiab] OR Syria*[tiab] OR Tunisia*[tiab] OR "United Arab Emirates"[tiab] OR UAE[tiab] OR Yemen*[tiab]) | 338 |
| **WOS** | ("medical student*" OR "medical undergraduate*" OR "undergraduate medicine" OR "medical intern*" OR "intern doctor*" OR "intern physician*" OR "medical resident*" OR "resident doctor*" OR "resident physician*") AND (migrat* OR emigrat* OR immigrat* OR "brain drain" OR "working abroad" OR "work abroad" OR "studying abroad" OR "study abroad" OR "training abroad" OR "train abroad" OR "intention to leave" OR "stay intention" OR "stay intentions" OR "career intention" OR "career intentions" OR "future career" OR workforce OR "leaving the country" OR "work overseas" OR "working overseas") AND (Arab* OR "Middle East" OR "North Africa" OR MENA OR Algeria* OR Bahrain* OR Comoros OR Djibouti OR Egypt* OR Iraq* OR Jordan* OR Kuwait* OR Lebanon OR Lebanese OR Libya* OR Mauritania* OR Morocco OR Moroccan OR Oman* OR Palestin* OR Qatar* OR "Saudi Arabia" OR Saudi OR KSA OR Somalia* OR Sudan* OR Syria* OR Tunisia* OR "United Arab Emirates" OR UAE OR Yemen*) | 151 |
| **Scopus** | ("medical student*" OR "medical undergraduate*" OR "undergraduate medicine" OR "medical intern*" OR "intern doctor*" OR "intern physician*" OR "medical resident*" OR "resident doctor*" OR "resident physician*") AND (migrat* OR emigrat* OR immigrat* OR "brain drain" OR "working abroad" OR "work abroad" OR "studying abroad" OR "study abroad" OR "training abroad" OR "train abroad" OR "intention to leave" OR "stay intention" OR "stay intentions" OR "career intention" OR "career intentions" OR "future career" OR workforce OR "leaving the country" OR "work overseas" OR "working overseas") AND (Arab* OR "Middle East" OR "North Africa" OR MENA OR Algeria* OR Bahrain* OR Comoros OR Djibouti OR Egypt* OR Iraq* OR Jordan* OR Kuwait* OR Lebanon OR Lebanese OR Libya* OR Mauritania* OR Morocco OR Moroccan OR Oman* OR Palestin* OR Qatar* OR "Saudi Arabia" OR Saudi OR KSA OR Somalia* OR Sudan* OR Syria* OR Tunisia* OR "United Arab Emirates" OR UAE OR Yemen*) | 167 |
| **Embase** | (exp medical student/ OR exp medical resident/ OR exp internship/ OR (medical student* OR medical undergraduate* OR undergraduate medicine OR medical intern* OR intern doctor* OR intern physician* OR medical resident* OR resident doctor* OR resident physician*).ti,ab,kw.) AND (exp brain drain/ OR exp migration/ OR exp emigration/ OR exp immigration/ OR exp career/ OR (migrat* OR emigrat* OR immigrat* OR brain drain OR working abroad OR work abroad OR studying abroad OR study abroad OR training abroad OR train abroad OR intention to leave OR stay intention* OR career intention* OR future career OR workforce OR leaving the country OR work overseas OR working overseas).ti,ab,kw.) AND (exp Middle East/ OR exp North Africa/ OR (Arab* OR Middle East OR North Africa OR MENA OR Algeria* OR Bahrain* OR Comoros OR Djibouti OR Egypt* OR Iraq* OR Jordan* OR Kuwait* OR Lebanon OR Lebanese OR Libya* OR Mauritania* OR Morocco OR Moroccan OR Oman* OR Palestin* OR Qatar* OR Saudi Arabia OR Saudi OR KSA OR Somalia* OR Sudan* OR Syria* OR Tunisia* OR United Arab Emirates OR UAE OR Yemen*).ti,ab,kw.) | 261 |

**Table S2.** Risk of Bias of the included studies using the simplified version of the Newcastle Ottawa Scale (NOS-xs2) for prevalence studies.

|  | **Risk of bias domains** | | |
| --- | --- | --- | --- |
|  | **Study sample selection** | | **Assessment of outcome** |
| **Study ID** | **1. Representativeness of the study sample** | **2. Sample size** | **3. Assessment of the outcome** |
| Abd El-Ghaffar & Abd El Salam, 2026 | b) Somewhat representative of the target population (non-random sampling). | a) Justified and satisfactory. | d) No description of the assessment tool. |
| Aboudeif et al., 2024 | b) Somewhat representative of the target population (non-random sampling). | a) Justified and satisfactory. | d) No description of the assessment tool. |
| Abukmail & Albarqouni, 2021 | d) No description of the sampling strategy. | b) Not justified or unsatisfactory. | b) Acceptable assessment tool. |
| Ahmed et al., 2025 | b) Somewhat representative of the target population (non-random sampling). | a) Justified and satisfactory. | b) Acceptable assessment tool. |
| Akl et al., 2008 | b) Somewhat representative of the target population (non-random sampling). | b) Not justified or unsatisfactory. | d) No description of the assessment tool. |
| Al Hadhrami et al., 2024 | b) Somewhat representative of the target population (non-random sampling). | a) Justified and satisfactory. | d) No description of the assessment tool. |
| Al-Beitawi et al., 2021 | b) Somewhat representative of the target population (non-random sampling). | b) Not justified or unsatisfactory. | d) No description of the assessment tool. |
| Al-Samarrai and Jadoo, 2018 | b) Somewhat representative of the target population (non-random sampling). | b) Not justified or unsatisfactory. | b) Acceptable assessment tool. |
| Alfadul et al., 2025 | b) Somewhat representative of the target population (non-random sampling). | a) Justified and satisfactory. | d) No description of the assessment tool. |
| Alfadul et al., 2026 | b) Somewhat representative of the target population (non-random sampling). | b) Not justified or unsatisfactory. | d) No description of the assessment tool. |
| Alfakhry et al., 2025 | b) Somewhat representative of the target population (non-random sampling). | b) Not justified or unsatisfactory. | d) No description of the assessment tool. |
| Alfiqi et al, 2026 | b) Somewhat representative of the target population (non-random sampling). | a) Justified and satisfactory. | b) Acceptable assessment tool. |
| Alsuhaibani et al., 2018 | b) Somewhat representative of the target population (non-random sampling). | b) Not justified or unsatisfactory. | d) No description of the assessment tool. |
| Ayman et al, 2025 | b) Somewhat representative of the target population (non-random sampling). | a) Justified and satisfactory. | b) Acceptable assessment tool. |
| Barnett-Vanes et al., 2016 | b) Somewhat representative of the target population (non-random sampling). | b) Not justified or unsatisfactory. | d) No description of the assessment tool. |
| Boughzala et al., 2019 | b) Somewhat representative of the target population (non-random sampling). | b) Not justified or unsatisfactory. | b) Acceptable assessment tool. |
| Darwish et al., 2026 | b) Somewhat representative of the target population (non-random sampling). | a) Justified and satisfactory. | d) No description of the assessment tool. |
| Deeb et al., 2026 | b) Somewhat representative of the target population (non-random sampling). | a) Justified and satisfactory. | d) No description of the assessment tool. |
| Fouad et al., 2015 | b) Somewhat representative of the target population (non-random sampling). | b) Not justified or unsatisfactory. | b) Acceptable assessment tool. |
| Gado et al., 2025 | b) Somewhat representative of the target population (non-random sampling). | a) Justified and satisfactory. | d) No description of the assessment tool. |
| Gismalla et al., 2017 | b) Somewhat representative of the target population (non-random sampling). | b) Not justified or unsatisfactory. | b) Acceptable assessment tool. |
| Haouari et al., 2024 | d) No description of the sampling strategy. | b) Not justified or unsatisfactory. | b) Acceptable assessment tool. |
| Kabbash et al., 2021 | b) Somewhat representative of the target population (non-random sampling). | a) Justified and satisfactory. | b) Acceptable assessment tool. |
| Lafta et al., 2018 | b) Somewhat representative of the target population (non-random sampling). | a) Justified and satisfactory. | b) Acceptable assessment tool. |
| Mohamed et al., 2015 | b) Somewhat representative of the target population (non-random sampling). | a) Justified and satisfactory. | b) Acceptable assessment tool. |
| Omar et al., 2023 | b) Somewhat representative of the target population (non-random sampling). | b) Not justified or unsatisfactory. | b) Acceptable assessment tool. |
| Sawaf et al., 2018 | b) Somewhat representative of the target population (non-random sampling). | b) Not justified or unsatisfactory. | b) Acceptable assessment tool. |
| Schumann et al., 2025 | b) Somewhat representative of the target population (non-random sampling). | a) Justified and satisfactory. | b) Acceptable assessment tool. |
| Soqia et al., 2024 | b) Somewhat representative of the target population (non-random sampling). | a) Justified and satisfactory. | b) Acceptable assessment tool. |
| Suboh et al., 2025 | b) Somewhat representative of the target population (non-random sampling). | a) Justified and satisfactory. | d) No description of the assessment tool. |
| Tabib et al., 2022 | d) No description of the sampling strategy. | b) Not justified or unsatisfactory. | d) No description of the assessment tool. |

**Table S3.** Reported push, pull, and mooring factors influencing migration intentions among Arab medical trainees.

| **Study ID** | **Pull factors** | **Push factors** | **Mooring factors** |
| --- | --- | --- | --- |
| Abukmail & Albarqouni, 2021 | NR | Societal expectations | NR |
| Ahmed et al., 2025 | Better personal lifestyle, looking for advanced training, better professional opportunities, better salary and working conditions | Avoid war and conflict | To be with family and friends, familiarity with the healthcare system, responsibility to help the country, positions available locally are better than those available abroad, preference for the lifestyle locally |
| Akl et al., 2008 | NR | NR | Marriage |
| Al-Beitawi et al., 2021 | NR | NR | Male gender |
| Al-Samarrai and Jadoo, 2018 | Opportunity to provide family with a better future, social, academic achievement, security, tourism, financial | Security concerns | Graduation certificate, availability of sufficient funds, parental consent |
| Alfadul et al., 2025 |  | Ongoing military conflict | Ongoing military conflict |
| Alfiqi et al, 2026 | Improve future career, financial conditions, better working conditions, more training and development opportunities, better clinical training, lifestyle, social conditions, personal reasons, independence, political conditions, family and friends | Exposure to workplace violence | NR |
| Ayman et al, 2025 | NR | Inadequate salaries, excessive workloads, administrative inefficiencies, verbal abuse, physical assault, negative media portrayal, lack of societal appreciation, feeling undervalued by the government, privacy violations, patients neglecting instructions, lack of workplace safety | NR |
| Boughzala et al., 2019 | Seeking a better quality of life, better working conditions, better training and supervision, satisfactory remuneration, personal and family fulfillment, practicing their profession to the best of their ability, valorization and social utility of the profession, good relationships with patients | Insufficient material resources and working conditions, insufficient salaries or income, high workload, insecurity in hospitals, demonization of doctors by the media, increase in the number of complaints and lawsuits against doctors, increasing patient demands, insufficient supervision and training, unclear status, non-existence of a college of family medicine | NR |
| Deeb et al., 2026 |  |  | Male gender, checkpoint burden |
| Fouad et al., 2015 | Access to advanced medical technologies, better lifestyle, better working environment, self-actualization, high salaries, more stable political circumstances, better professional stability, higher job satisfaction, desire to settle abroad, more job opportunities, obtaining citizenship of the destination country, presence of relatives abroad, better research opportunities and funding abroad, better working conditions of trainees abroad including financial situation and professional standards, better clinical training opportunities abroad, being inspired by successful experiences of doctors who traveled abroad, perception that Egyptian society values those with abroad training more highly, absence of training programs in Egypt for the specialty or subspecialty of choice | Low quality standards in the health sector, relatively low income of doctors, favoritism, low level of appreciation of doctors, harassment of doctors, political circumstances, lack of research funding, lack of good education for participants’ children, unavailability of the specialty of interest, more competition | The feeling of belonging and desire to serve the country, inability to leave family and friends, social factors, religious factors, and parental pressure |
| Gismalla et al., 2017 | Better facilities, international recognition, good and standard training, helping the community, role models, job opportunities, financial reasons, avoiding night calls | NR | Free hand training, social reasons, low cost, lack of competition, helping the community, role models, job opportunities, financial reasons, advice from family, avoiding night calls |
| Haouari et al., 2024 | NR | Dissatisfaction with working conditions, workplace safety, salaries, workload, supervision, the quality of hospital training, the national political situation, and social security | NR |
| Kabbash et al., 2021 | Better research opportunities | Inadequate salary, need to work extra time in private hospitals due to inadequate salary, lack of appreciation from the government, excessive workload or unreasonable work hours, lack of appreciation from society, negative media attitudes towards physicians, workplace violence including verbal abuse and physical assault | NR |
| Lafta et al., 2018 | Seeking a better personal lifestyle, seeking advanced training, seeking better professional opportunities, seeking better pay and working conditions, being with family | Avoiding war and conflict | Connections with families and friends, feelings of responsibility to assist Iraq, familiarity with the health services |
| Mohamed et al., 2015 | Professional advancement, better working conditions, financial reasons | Political reasons | NR |
| Omar et al., 2023 | Higher salaries, presence of better education, presence of a reasonable number of patients relative to that of residents, presence of a better lifestyle | Low salaries, poor education, and lower ranking of Jordan residency programmes | Leaving friends and relatives, acquiring travel visas, male gender, expatriate status, having relatives/friends in the destination country |
| Sawaf et al, 2018 | Training opportunities, clinical training quality and content, research training opportunities, learning in specialty programs, working conditions as a resident, financial status for residents, effect of residency training on work after residency, work opportunities, working conditions after residency, financial status for doctors after residency | Personal issues related to partner, parents, and children, social issues related to customs and traditions, safety issues related to the Syrian crisis | Acquiring a visa to the foreign country, required exams, costs of travelling, acquiring another nationality, male gender, having previous clinical training abroad, having relatives or friends living abroad who could assist |
| Schumann et al.,2025 | Career opportunities | Financial instability, lack of career opportunities, socio-political instability and lack of security | Social support and networks in the destination country |
| Soqia et al, 2024 | Greater opportunities for kids, greater opportunities for partner, opportunity to gain money, continuing education, safer and more stable life, preference for a certain type of life, quality of practical life | Lack of specialists | Ability to maintain social relationships, sense of belonging |
| Tabib et al, 2022 | NR | Dissatisfaction with the distribution of tasks and organization of work, safety at work, comfort, time allocated to personal life, salary, political, health and educational situation in the country was considered unsatisfactory | Male gender, having siblings abroad |

**Figure S1:** Forest plot of the prevalence of migration intention among Arab medical trainees: Subgroup analysis by training level

**Figure S2:** Forest plot of the prevalence of migration intention among Arab medical trainees: Subgroup analysis by gender

**Figure S3:** Forest plot of the prevalence of migration intention among Arab medical trainees: Subgroup analysis by region

**Figure S4:** Forest plot of the prevalence of migration intention among Arab medical trainees: Subgroup analysis by country income level

**Figure S5:** Forest plot of the prevalence of migration intention among Arab medical trainees: Subgroup analysis by fragility status

**Figure S6:** Forest plot of the prevalence of migration intention among Arab medical trainees: Subgroup analysis by type of migration intention

**Figure S7:** Forest plot of the prevalence of migration intention among Arab medical trainees: Subgroup analysis by sampling technique

**Figure S8:** Forest plot of the leave-one-out sensitivity analysis for the prevalence of migration intention among Arab medical trainees
